# Dihydroartemisinin inhibits vascular endothelial growth factor-induced endothelial cell migration by a p38 mitogen-activated protein kinase-independent pathway

**DOI:** 10.3892/etm.2014.1997

**Published:** 2014-09-30

**Authors:** LING GUO, FENGYUN DONG, YINGLONG HOU, WEIDONG CAI, XIA ZHOU, AI-LING HUANG, MIN YANG, THADDEUS D. ALLEN, JU LIU

**Affiliations:** 1 Laboratory of Microvascular Medicine, Medical Research Center, Shandong Provincial Qianfoshan Hospital, Shandong University, Jinan, Shandong 250014, P.R. China; 2 Department of Cardiology, Shandong Provincial Qianfoshan Hospital, Shandong University, Jinan, Shandong 250014, P.R. China; 3 Department of Emergency Medicine, Shandong Provincial Qianfoshan Hospital, Shandong University, Jinan, Shandong 250014, P.R. China; 4 Department of Traditional Chinese Medicine, Shandong Provincial Qianfoshan Hospital, Shandong University, Jinan, Shandong 250014, P.R. China; 5 Department of Nephrology, Shandong Provincial Qianfoshan Hospital, Shandong University, Jinan, Shandong 250014, P.R. China; 6 Department of Orthopedics, Xijing Hospital, The Fourth Military Medical University, Xi’an, Shaanxi 710032, P.R. China; 7 G.W. Hooper Research Foundation, University of California, San Francisco, CA 94143-0552, USA

**Keywords:** dihydroartemisinin, migration, p38 mitogen-activated protein kinase, endothelial cell, angiogenesis

## Abstract

Dihydroartemisinin (DHA), a semi-synthetic derivative of artemisinin, has been demonstrated to possess a strong antiangiogenic activity. However, the molecular mechanisms underlying this effect remain unclear. Endothelial cell (EC) migration is an essential component of angiogenesis, and the p38 mitogen-activated protein kinase (MAPK) signaling pathway plays a key role in the regulation of migration induced by vascular endothelial growth factor (VEGF). The aim of the present study was to investigate the effects of DHA on EC migration and the p38 MAPK signaling pathway. Human umbilical vein ECs (HUVECs) were treated with DHA and VEGF-induced migration was analyzed. The activation of p38 MAPK was detected by western blot analysis, and the migration assays were performed with a p38-specific inhibitor, SB203850. It was revealed that 20 μM DHA significantly reduced EC migration in the transwell migration assay, wound healing assay and electrical cell-substrate impedance sensing real-time analysis. However, DHA did not affect p38 MAPK phosphorylation or expression. In the absence or presence of SB203850, DHA induced a similar proportional reduction of EC migration in the three migration assays. Therefore, the present study demonstrated that DHA inhibits VEGF-induced EC migration via a p38 MAPK-independent pathway.

## Introduction

Artemisinin is a sesquiterpene lactone isolated from the *Artemisia annua* plant that has been used extensively as an antimalarial drug (1). Dihydroartemisinin (DHA) is the active metabolite of artemisinin compounds, and a semisynthetic derivative of artemisinin (2). DHA is an effective, water-soluble antimalarial drug, with fewer side-effects compared with other drugs (3). In addition, DHA exhibits strong antitumor and antiangiogenesis effects (4). However, the underlying mechanisms of DHA remain poorly understood.

Angiogenesis, the formation of new capillaries branching from existing vessels, plays a critical role in embryonic development, wound-healing and the menstrual cycle (5). Abnormal angiogenesis is associated with tumor growth, diabetes, rheumatoid arthritis and atherosclerosis (6). Under hypoxic conditions, tumor cells or other growing cells secrete growth factors, including vascular endothelial growth factor (VEGF) and basic fibroblast growth factor (bFGF). These growth factors activate endothelial cells (ECs) in nearby capillaries, leading to the migration of ECs branching out of the vessel (7). The cells proliferate and differentiate to form a network of new capillaries. It has been well established that solid tumor growth requires a neovascular network, which enables rapid proliferation of tumor cells by providing oxygen and nutrients (8).

EC migration is an essential component of angiogenesis (7). This motile process is regulated by chemotactic, haptotactic and mechanotactic stimuli (7). Typically, chemotaxis of ECs is driven by growth factors, including VEGF and bFGF, whereas haptotaxis is associated with increased EC motility in response to integrins binding to the extracellular matrix (5). The process requires the activation of several signaling pathways that converge on cytoskeletal remodeling (9).

Mitogen-activated protein kinase (MAPK) pathways constitute a large network of signaling cascades that regulate diverse physiological processes, including cell migration (10). The functions of MAPKs are mediated through the phosphorylation of substrates, including phospholipases, transcription factors and cytoskeletal proteins (11). To date, three major subfamilies of MAPKs have been well-characterized: Extracellular signal-regulated kinases (ERKs), p38 MAPKs and c-Jun N-terminal kinases (JNKs). The p38 pathway conveys the VEGF signal to microfilaments, inducing rearrangements in the actin cytoskeleton that regulate cell migration (12). Through the modulation of cell migration, p38 MAPK is an important regulator of angiogenesis (13).

In the present study, DHA was hypothesized to inhibit EC migration via the p38 MAPK pathway. Cell migration assays were performed on human umbilical vein ECs (HUVECs) with the addition of DHA, and the phosphorylation of the p38 MAPK was examined by western blot analysis. The role of p38 MAPK was further examined using an electrical cell-substrate impedance sensing (ECIS) system with the specific inhibitor, SB203850.

## Materials and methods

### Cell culture

HUVECs were purchased from the American Type Culture Collection (Manassas, VA, USA) and maintained in endothelial growth medium (EGM-2), supplemented with the EGM-2-MV bullet kit (Lonza, Basel, Switzerland) and antibiotics (100 IU/ml penicillin and 100 μg/ml streptomycin), in a humidified atmosphere at 37°C and 5% CO_2_. DHA (sc-211332; Santa Cruz Biotechnology, Inc., Santa Cruz, CA, USA), anisomycin (sc-3524, Santa Cruz Biotechnology, Inc.) and SB203850 (Cell Signaling Technology, Inc., Beverly, MA, USA) were dissolved in dimethyl sulfoxide (DMSO).

### Boyden chamber migration assay

Cell migration assays were performed using modified 24-well Boyden chambers (Costar, Acton, MA, USA), containing a polycarbonate membrane with 8.0-μm pores. HUVECs were starved in basic EGM-2 (serum/growth factor free) overnight at 37°C, prior to being harvested with trypsin and resuspended in basic EGM-2. The single cell suspensions with 20 μM DHA or 20 μM SB203850 were seeded at 1×10^5^ cells/well in the upper chamber, while 0.5 ml EGM-2 with 20 ng/ml VEGF was added to the bottom chamber as chemoattractants. After 24 h incubation, the migrated cells on the bottom surface were stained with 0.1% crystal violet (Santa Cruz Biotechnology, Inc.) and counted under an Olympus LCX100 Imaging system (Olympus Corporation, Tokyo, Japan).

### Wound healing migration assay

HUVECs were grown to confluence in 24-well plates and starved for 2 h. The media were changed to basic endothelial growth basal medium (EBM-2), supplemented with 100 ng/ml VEGF, and a scratch (wound) was made across the monolayer using a sterile pipette tip. DHA was added to the culture medium with a final concentration of 20 μM. Images of the wells were captured at fixed points to record the area of clearing at time 0 and 8 h, and ImageJ software (NIH, Bethesda, MD, USA) was used to quantitate the cleared area.

### Western blot analysis

HUVECs treated with 20μM DHA were collected at different time points. To generate a positive control for p38 MAPK activation, a group of HUVECs were treated with 1 μg/ml anisomycin for 1 h. Cell lysates were prepared in radioimmunoprecipitation assay buffer [20 mM Tris (pH 7.5), 150 mM NaCl, 50 mM NaF, 1% NP-40, 0.1% deoxycholate, 0.1% SDS and 1 mM EDTA] (Santa Cruz Biotechnology, Inc.), supplemented with 1 mM phenylmethylsulfonyl fluoride and 1 μg/ml leupeptin (Santa Cruz Biotechnology, Inc.). Cleared cell lysates were subjected to SDS-PAGE using 10% polyacrylamide gel and transferred to polyvinylidene fluoride membranes. Membranes were blocked with 2.5% non-fat milk, and incubated with primary antibodies at 4°C overnight in phosphate-buffered saline Tween-20 (Santa Cruz Biotechnology, Inc.). The primary antibodies used were total-p38, phospho-p38 (Cell Signaling Technology, Inc.) and β-actin (Sigma-Aldrich, St. Louis, MO, USA). Immunoreactivity was visualized with horseradish peroxidase-conjugated secondary antibodies and an enhanced chemiluminescence reagent (Santa Cruz Biotechnology, Inc.). The blots were analyzed using a Bio-Rad imaging system (Bio-Rad, Hercules, CA, USA).

### ECIS migration analysis

Real-time EC migration was measured using the ECIS technique (ECIS model 1600; Applied BioPhysics, Troy, NY, USA). Briefly, eight-well ECIS arrays (8W10E+) were coated with fibronectin (Invitrogen Life Technologies, Carlsbad, CA, USA). HUVECs were plated at a confluent density to form monolayers directly on top of the electrodes. Next, an elevated voltage pulse of 40 kHz was applied to the arrays for 30 sec, which resulted in the death and detachment of cells from the electrodes. As a result, the wound was healed by the surrounding cells. Following treatment with DHA or SB203850, an alternating current was applied to the cells across the electrodes and the electrical resistance was recorded. Data plots are representative of triplicate experiments, with each graph showing the resistance readings from a separate well, at 40 distinct electrodes per well.

### Statistical analysis

Statistically significant differences were assessed using a paired-samples t-test. All statistical analyses were performed using SPSS 19.0 statistical software (Chicago, IL, USA) with a significance level of P<0.05.

## Results

### DHA inhibits EC migration

**Figure 1:**
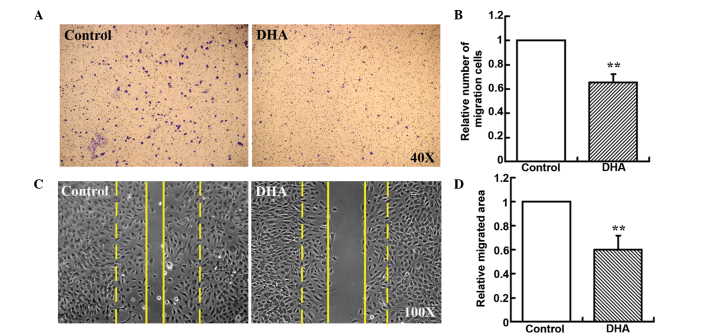
Cell migration of human umbilical vein endothelial cells treated with DHA. (A) Representative cell staining images following the transwell migration assay. (B) Number of cells migrating through the transwell membrane (n=6); ^**^P<0.01, vs. control. (C) Representative images of the wound healing assay. Dashed line indicates 0 h and the solid line indicates 8 h. (D) Migrated area following the wound healing assay (n=12); ^**^P<0.01, vs. control. DHA, dihydroartemisinin.

Boyden chamber-type cell migration assays were used to assess the effect of DHA on EC migration. A low concentration of 20 μM DHA was used, since it had been demonstrated to be sufficient to inhibit angiogenesis *in vitro* (14). The number of HUVECs migrating across the polycarbonate membrane was significantly reduced in the groups treated with 20 μM DHA (33.76%, P<0.01; Fig. 1A and B). Cell migration during *in vitro* wound healing mimics the process of EC motility *in vivo* (12,15). Thus, wound healing migration assays were also performed, and the migrated area of HUVECs was significantly reduced in the DHA-treated groups when compared with the groups treated with vehicle DMSO alone (40.97%, P<0.01; Fig. 1C and D). Thus, these *in vitro* assays indicated that DHA induced the reduction of EC migration.

### Activation of p38 MAPK is unaffected by DHA in ECs

**Figure 2:**
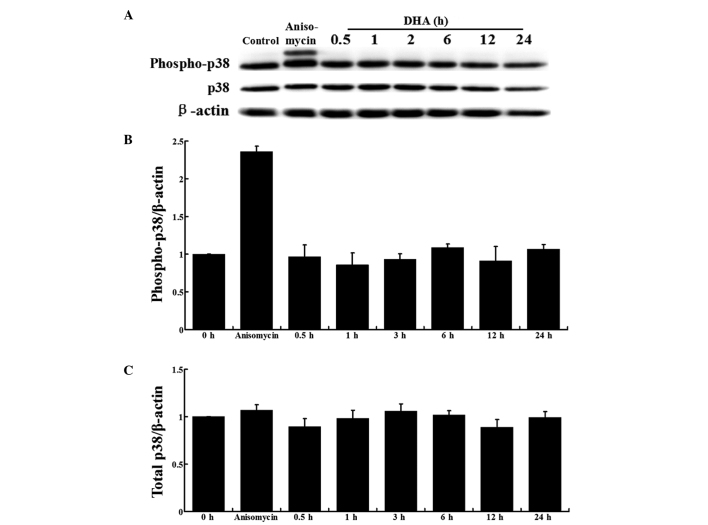
Effect of DHA on the activation of p38 mitogen-activated protein kinase (MAPK) in human umbilical vein endothelial cells (HUVECs). (A) Immunoblots of phospho-p38 MAPK, p38 MAPK and β-actin from protein samples of HUVECs treated with DHA at various time points. Densitometric analysis of the blots of (B) phospho-p38 MAPK and (C) total p38 MAPK. DHA, dihydroartemisinin.

In ECs, p38 MAPK activation by VEGF mediates actin reorganization and cell migration (12). To examine the effects of DHA on p38 MAPK activation, HUVECs cultured in EBM-2, containing 100 ng/ml VEGF, were treated with 20 μM DHA and the protein expression was analyzed at different time points. HUVECs treated with anisomycin, a known activator of p38 MAPK, were used as a positive control (16). Western blot analysis showed that the total p38 and phospho-p38 MAPK protein expression levels remained unchanged during DHA treatment at all the indicated time points (Fig. 2A). Densitometric analysis further confirmed that DHA did not affect p38 MAPK activation or expression (Fig. 2B and C).

### DHA-induced repression of EC migration is unaffected by SB203850

**Figure 3:**
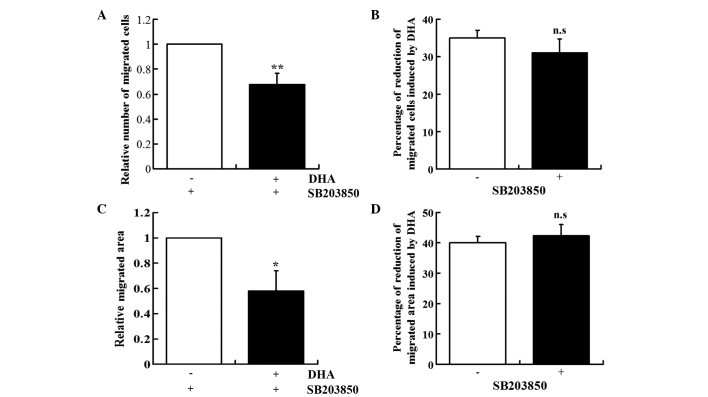
Effect of SB203850 on DHA-induced inhibition of endothelial cell migration. (A) Transwell migration assay of human umbilical vein endothelial cells (HUVECs) treated with SB203850 and DHA (n=6); ^**^P<0.01. (B) Percentage reduction of migrated cells induced by DHA in the absence or presence of SB203850. (C) Wound healing assay of HUVECs treated with SB203850 and DHA (n=12); ^*^P<0.05. (D) Percentage reduction in the migrated area induced by DHA in the absence or presence of SB203850. n.s., non-significant. DHA, dihydroartemisinin.

SB203850 is a pyridinyl imidazole derivative that inhibits p38 MAPK phosphorylation, but does not reduce p38 MAPK expression (17). The role of p38 MAPK in mediating the DHA-induced inhibition of EC migration was ascertained by examining the effect of SB203580 on the DHA-treated HUVECs. In the presence of SB203580, DHA also induced the reduction of EC migration in the Boyden chamber assay (30.38%, P<0.01) and wound healing assay (43.04%, P<0.05; Fig. 3A and C). The levels of reduction induced by DHA in the two assays were similar in the absence or presence of SB203850 (Fig. 3B and D).

**Figure 4:**
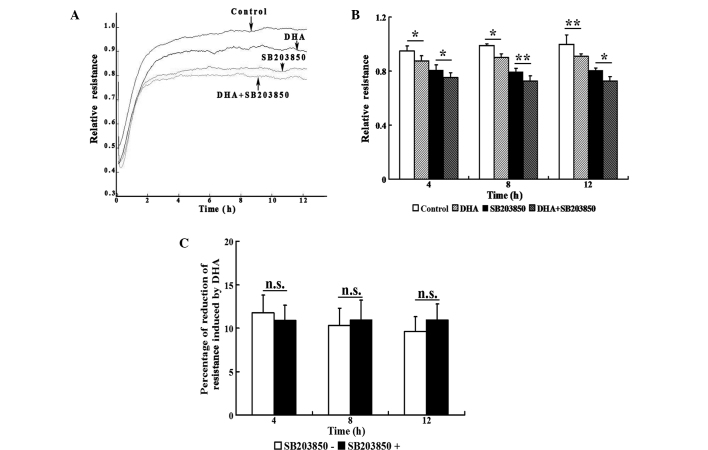
Transendothelial resistance determined using an electrical cell-substrate impedance sensing wound healing assay in human umbilical vein endothelial cells (HUVECs) treated with DHA and SB203850. (A) Real-time resistance measurement of the HUVEC monolayer treated with DHA and/or SB203850. (B) Bar graph showing the mean percentage of transendothelial resistance (n=4); ^*^P<0.05; ^**^P<0.01. (C) Percentage reduction of resistance induced by DHA in the absence or presence of SB203850 (n=4). n.s., non-significant. DHA, dihydroartemisinin.

To capture the subtle alterations of cell migration, an ECIS system was implemented, which allowed for real-time measurements of the resistance caused by the migration of ECs. The transendothelial resistance was significantly reduced when HUVECs were treated with 20 μM DHA (P<0.05; Fig. 4A and B). Treatment with 20 μM SB203580 also significantly decreased the transendothelial resistance (P<0.05); however, treatment with DHA and SB203850 induced an additional reduction in transendothelial resistance at 4, 8 and 12 h (Fig. 4A and B). In the absence or presence of SB203850, the reduction of transendothelial resistance induced by DHA showed no statistically significant difference at any of the time points (Fig. 4C). Therefore, the inhibitory effects of DHA on EC migration are not mediated by the p38 MAPK pathway.

## Discussion

Antiangiogenic therapy targeting ECs to block neovascularization has become an effective anticancer strategy (18). DHA, a widely used antimalarial drug with minimal side-effects, has been reported to be highly effective against tumor angiogenesis (14). The aim of the present study was to investigate the mechanisms underlying the effect of DHA on EC migration, a key process in angiogenesis. DHA was shown to inhibit EC migration in three types of migration assays. In addition, the role of the p38 MAPK signaling pathway in the effect of DHA was further investigated, and p38 MAPK activation was shown to not be affected by DHA. Furthermore, the p38 MAPK inhibitor, SB203850, did not inhibit or augment the DHA-induced reduction of EC migration. To the best of our knowledge, the present study is the first to investigate the signaling transduction pathways mediating the effects of the artemisinin family of drugs on EC migration.

EC migration is a dynamic and multistep process that plays a crucial role in the initiation and progression of angiogenesis (19). Previous studies have demonstrated the antiangiogenetic effects of DHA and other artemisinin derivatives (20,21). DHA has been shown to inhibit HUVEC migration in a dose-dependent manner (14), and also to reduce the migration of murine lymphatic ECs (22). In the current study, HUVEC migration was examined through Boyden transwell assays, wound healing assays and ECIS analysis. Results from all three methods confirmed the inhibitory role of DHA on EC migration, which may significantly contribute towards the antiangiogenetic effects.

Initiation of cell migration in response to cytokines, including VEGF, is a highly controlled process requiring the co-operation of signaling pathways, involving the extracellular matrix, transmembrane receptors, such as integrins, and actin cytoskeleton-associated motile apparatus (23,24). MAPK pathways are important components of the signaling network, transducing the migratory signals generated by VEGF and playing an important role in the regulation of angiogenesis (12). VEGF activates the p38 MAPK pathway via KDR/Flk-1 in ECs, while SB203580 induces the inhibition of p38 activity and cell migration (12). Artemisinin and its derivatives have been shown to increase or suppress p38 MAPK activity in different experimental settings (25,26). In the present study, DHA was shown to not affect the VEGF-induced activation of p38 MAPK, indicating that DHA exerts antimigratory effects independent of the p38 MAPK pathway.

The ECIS system provides a highly sensitive method to monitor real-time cell migration (27). Consistent with previous observations, ECIS analysis revealed that SB203850 significantly inhibited cell migration. However, the application of DHA to SB203850-treated HUVECs resulted in an additional decrease in cell migration. Thus, inhibiting the p38 MAPK pathway failed to prevent the DHA-induced reduction of cell migration. These results further confirm that p38 MAPK does not mediate the DHA-induced inhibition of EC migration.

Although p38 MAPK plays a key role in promoting cell migration, other signaling pathways are also involved in regulating this process. The nuclear factor-κB (NF-κB) family comprises a wide range of transcription factors that are involved in numerous cellular functions (28). Activation of the NF-κB pathway promotes cell migration. Extracellular stimuli, such as high glucose concentrations, inhibit EC migration via the suppression of the NF-κB pathway (29). DHA inhibits the NF-κB pathway by preventing the nuclear translocation of the p65/p50 complex (30). In addition, the protein kinase B (PKB)signaling pathway positively regulates cell migration, and oxidized low-density lipoprotein inhibits EC migration through the suppression of this pathway (31). Artemisinin and its derivatives have been shown to inhibit PKB activity (32). Therefore, the DHA-induced inhibition of EC migration may be mediated by these p38-independent signaling pathways.

In conclusion, the results of the present study demonstrated that DHA inhibits EC migration, which is independent of p38 MAPK signaling. These observations provide an insight into the mechanisms underlying the antiangiogenetic effects of the artemisinin family of drugs, which may develop into therapeutic agents against cancer and other vascular-associated diseases.
